# Estrogenic Isoflavones in Clover Plants, Flower Nectar, Unripe Honeys and Mature Honeys: A Natural Biochemical Transformation of Isoflavones by Honeybees

**DOI:** 10.3390/foods13111739

**Published:** 2024-06-01

**Authors:** Sharmin Sultana, Kevin J. Foster, Ivan Lozada Lawag, Lee Yong Lim, Katherine Hammer, Cornelia Locher

**Affiliations:** 1 Division of Pharmacy, School of Allied Health, University of Western Australia, Perth 6009, Australia; sharmin.sultana@research.uwa.edu.au (S.S.); ivan.lawag@uwa.edu.au (I.L.L.); lee.lim@uwa.edu.au (L.Y.L.); 2 School of Agriculture and Environment, University of Western Australia, Crawley 6009, Australia; 3 School of Biomedical Sciences, University of Western Australia, Perth 6009, Australia; katherine.hammer@uwa.edu.au; 4 Cooperative Research Centre for Honeybee Products Limited, 128 Yanchep Beach Road, Perth 6035, Australia

**Keywords:** phytoestrogen, red clover, sainfoin, purple clover, nectar, honey, high-performance thin-layer chromatography (HPTLC)

## Abstract

This study is the first to report on the presence of oestrogenic compounds in different clover flower nectar samples, in bee-deposited nectars collected from hive combs (unripe honey) and in mature honeys harvested from the same hives. The clover species investigated were two red clover (*Trifolium pratense*) cultivars, bred specifically for high isoflavone content, alongside a sainfoin (*Onobrychis viciifolia*) and a purple clover (*T. purpureum*) cultivar. A total of eight isoflavones, four of them non-glycosidic (biochanin A, formononetin, genistein and daidzein) the others glycosidic (sissotrin, ononin, genistin and daidzin), were targeted for identification and quantification in this study using high-performance thin-layer chromatography (HPTLC). Leaves and flower bracts of the clover samples were also investigated. Different isoflavone profiles were found across the four clover species and also in the different samples collected from each species indicating that, most likely due to the activity of honeybee (*Apis mellifera*) salivary enzymes, biochemical conversions take place when these bioactive compounds transition from flower nectar into ripe honey. Among the four investigated clover species, the two red clover cultivars, including their honeys, were found to contain higher levels of estrogenic compounds compared to other two cultivars.

## 1. Introduction

Clovers belong to the genus *Trifolium* and comprise approximately 300 species of flowering plants in the legume family Fabaceae. Originating in Europe and North America and the Mediterranean, clovers are small herbaceous plants that can be annual, biennial or short-lived perennials. They typically grow up to 30 cm tall [1]. Their leaves are trifoliate, meaning they have three leaflets and their flowers form heads or dense spikes of various colors, including red, purple, white and yellow. 

Clovers adapt well to various soils and climates and are extensively cultivated as fodder plants. They are sown alone or mixed with ryegrass for silage production, as they grow freely, rebound after mowing and provide abundant and nutritious feed for livestock. Moreover, clovers have the ability to fix nitrogen in the soil, thus reducing the use of fertilizers [1]. The most widely cultivated clover species are red clover (*Trifolium pratense* L.) and white clover (*T. repens*) along with purple clover (*T. purpureum*) and sainfoin (*Onobrychis viciifolia*), which is also known as ‘holy clover’.

Clover blossoms produce copious amounts of nectar and are highly attractive to pollinating insects, particularly honeybees (*Apis mellifera*), which are their most efficient pollinators [2,3]. Clover honey is considered a premium product [4]; thus, apiculture on clover pastures is a common complementary agricultural activity [5]. It presents a win–win situation as farmers benefit from increased reseeding due to bee pollination while clover blooms serve as an attractive nectar source for beekeepers [5].

Clover honey tends to be rich in phenolic acids and flavonoids [6], which contribute to a wide range of biological effects. However, despite the potential benefits of clovers for agriculture and apiculture, many clovers species have not yet been assessed for the quality of their pollen or honey, and most remain unassessed for their bioactivities and thus their potential as medicinal honeys [6]. 

There is an increasingly positive market trend towards honey products with medicinal benefits for the retail and pharmaceutical industry following the success of New Zealand and Australian manuka honeys. Red clover in particular has been recognized for its positive impacts on human health, specifically its role as phytoestrogen in the treatment of menopausal symptoms [7,8]. To date, approximately 40 different isoflavones have been reported from red clover flower heads [9,10], the most abundant being biochanin A and formononetin, along with lower concentrations of daidzein, glycitein and genistein [11,12,13,14]. Other isoflavones found in the leaves of red clover include calycosin, prunetin, afrormosin, texasin, irilin B, irilone and pseudobaptigenin [15,16,17] alongside other flavonoids, such as quercetin and kaempferol and other phenolics, phenolic acids in particular (e.g., caffeic, rosmarinic and chlorogenic acid) [18]. 

Among the reported isoflavones, two stand out as more potent in terms of the form of estrogenic isoflavonoids, genistein and daidzein [19,20,21,22]. Other isoflavones found in red clover are chemically related; biochanin A and formononetin, for example, are methylated precursors of genistein and daidzein. These four isoflavones can also exist as glycosides, with sissotrin and ononin as the glucosides of biochanin A and formononetin, respectively, and genistin and daidzin as glucosides of genistein and daidzein, respectively.

While research on the phytochemical composition of various honeys is growing, studies focusing on clover honeys and their bioactivities have been limited. To date, there appears to be no study that has focused specifically on the presence of phytoestrogenic isoflavonoids in clover honeys. The popularity of red clover leaf and flower extracts for the management of post-menopausal symptoms, such as hot flushes, night sweats, vaginal dryness and insomnia, which is based on their phytoestrogenic isoflavonoid content, has created the impetus for this study. In order to explore potential future opportunities for both the pharmaceutical and apiculture industries, the study aims to identify and quantify different phytoestrogenic isoflavones in leaves and flower brackets as well as in the respective flower nectars and honeys of four different clover species, two red clover (*Trifolium pratense*) cultivars, bred specifically for high isoflavone content, alongside a sainfoin (*Onobrychis viciifolia*) and a purple clover (*T. purpureum*) cultivar.

## 2. Materials and Methods

### 2.1. Plant Establishment and Growing Conditions

**Figure 1:**
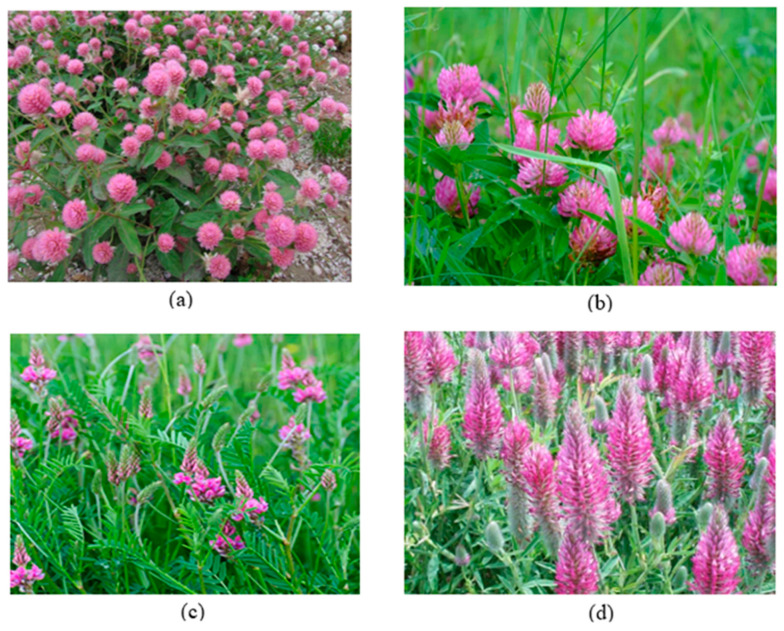
Clover cultivars: (**a**) NSE, (**b**) NFE, (**c**) Othello and (**d**) Electra.

Clover plants were grown in enclosed shade houses (10 m × 4 m) at the University of Western Australia’s Shenton Park Field Station in 2021 and 2022 (Figure 1). The cultivars used were Electra (purple clover), Othello (sainfoin clover) and two patented red clover cultivars, referred to as NSE and NFE. Each clover species was sown in a seedling tray containing 30 peat pots and covered lightly with potting mix. Three days after sowing, appropriate rhizobium inoculant was applied by watering can, by adding 40 mL of inoculum to 10 L of water (one watering can for five trays). Peat pots were watered daily. Seedlings were transferred to outside benches for acclimatization after five weeks in the glasshouse. Prior to transplanting to the shade house, seedlings were thinned randomly to one per peat pot. All clovers were transplanted into their plots in the enclosed shade houses at 30 cm spacing. Soluble fertilizer (Thrive^®^ All Purpose; 5 g per 10 L) was applied to all seedlings after transplanting (1.5 L per m^2^). Using micro jets (Pope half-circle Veri-flow), irrigation was supplied to each plant for 20 min through 19 mm premium black poly pipe (Holman, Perth, Western Australia) when required. Plants were fertilized by hand with single superphosphate with potash (6.8% P, 12.4% K and 8.3% S) prior to flowering.

The purpose of the enclosed shade house plots was to collect monofloral honeys from a hive of bees foraging in each respective enclosure. Each enclosed shade house had a nucleus beehive placed when half of all plants were in their flowering stage. This approach allowed to determine the phytochemical characteristics of each clover honey without potential interference from co-flowering species as might be the case for wild-harvested honeys. The nucleus beehives were checked regularly, frames with capped honey were transported to the laboratory, and unripe and ripe honeys were then manually removed from the frames.

### 2.2. Sample Collection

#### 2.2.1. Clover Leaves and Flower Bracts

Clover leaves, with their trifoliate arrangement, typically consist of three leaflets. The term ‘flower bracts’ refers to the structures that cradle individual flowers within a larger inflorescence. Leaves and flower bracts were manually collected using tweezers. The freshly picked samples were placed in plastic bags and transported from the field station to the laboratory in cooler boxes and stored at 4 °C until analyzed.

#### 2.2.2. Flower Nectar

Researchers have devised various methods to extract nectar for analysis [23]. In response to the small flower size and associated low volumes of nectar encountered in this study, a washing method was selected to extract nectar [24]: Flowers were collected early in the morning at an outside temperature of around 17 °C, deposited into plastic bags and transported to the laboratory in cooler boxes. To ensure that the collected nectar was not contaminated by pollen, pollen-carrying anthers were removed from the flowers using a pair of scissors. Approximately 40 g of flower tops were then soaked in 250 mL of distilled water for 24 h at 4 °C. During this period, the nectar dissolved in the water, creating a nectar–water solution which was transferred into another glass container after straining using a stainless-steel strainer. The solution was stored at −80 °C until analysis. To quantify the amount of collected nectar per flower head, the average flower head weight of each species was determined from the individual weight of 20 samples.

#### 2.2.3. Unripe and Ripe Honey

Unripe honey was directly taken from the honeycombs, whereas extraction of mature honey required the manual uncapping of the cells using an anti-cutter (Keji, Guangzhou, China). The distinction between unripe and mature honey is crucial: Unripe honey may have higher water content and still requires further evaporation by bees through fanning their wings over the cells in the honeycomb, while mature honey is low in water content, thus self-preserving and as such ready for consumption. All collected honey samples were stored at −80 °C until analysis to prevent degradation and maintain quality.

### 2.3. Chemicals and Reagents

All reagents and solvents used in this study were of analytical grade. Trihydroxyflavone (THF) was obtained from Alfa Aesar (Heysham, UK); all other flavonoid standards were sourced from ChemFaces (Wuhan, China); methanol was purchased from Scharlau (Barcelona, Spain), and dichloromethane, ethyl acetate, glacial acetic acid and formic acid from Ajax Finechem (New South Wales, Australia). Silica gel 60 F_254_ HPTLC glass plates (20 cm × 10 cm) were purchased from Merck KGaA (Darmstadt, Germany). Vanillin was sourced from Chem Supply Australia Pty Ltd. (Port Adelaide, Australia), and sulfuric acid from PharmAust Manufacturing (Welshpool, Australia).

#### Reagent Preparation

A mixture of dichloromethane:ethyl acetate:acetic acid (12:1:12, *v*/*v/v*) was prepared as the mobile phase for the high-performance thin-layer chromatography (HPTLC) analysis of non-glycosidic isoflavones (mobile phase A), whereas ethyl acetate:methanol:glacial acetic acid:formic acid (11:1:1:1, *v*/*v/v/v*) was used as mobile phase for the detection and quantification of glycosidic isoflavones (mobile phase B). All isoflavonoid standards were prepared in a concentration of 0.5 mg/mL in methanol. Methanolic solution of THF (0.5 mg/mL) was used as internal reference standard for mobile phase A to confirm system suitability, whereas methanolic solution of Naringin (0.5 mg/mL) was used for mobile phase B. Vanillin in sulfuric acid (VSA) and natural product (NP) reagent (also known as Naturstoff reagent or Neu’s reagent, diphenylborinic acid 2-aminoethyl ester (DPBA) and 2-aminoethyl diphenylborinate) in combination with polyethylene glycol (PEG-400) were used as derivatisation reagents for mobile phase A and B, respectively. VSA was prepared by dissolving 1 g of vanillin and 2 mL of sulfuric acid in 100 mL of methanol. NP was prepared by dissolving 1 g of 2-aminoethyl diphenylborinate in methanol before the volume of the solution was made up to 100 mL (1% *m*/*v*) [25]. PEG reagent was prepared by mixing 5 g of polyethylene glycol in ethanol and then the volume of the solution was adjusted to 100 mL (5% *m*/*v*) [25].

### 2.4. Sample Preparation

#### 2.4.1. Leaves and Flower Bracts

Methanolic solutions of clover leaves and flower bract samples were prepared by crushing the fresh plant material in a mortar using a pestle. The content in the mortar was then transferred into benchtop centrifuge tubes, thoroughly washed with methanol and brought to a final concentration of 10 mg of fresh plant material per mL of methanol for subsequent HPTLC analysis.

#### 2.4.2. Flower Nectar

The aqueous nectar solution was freeze-dried (Alpha 1-2 LDplus Freeze dryer, Martin Christ GmbH, Osterode am Harz, Germany), and the resulting dried nectars reconstituted in distilled water at a concentration of 0.5 g/mL. The aqueous nectar solution was then extracted three times with 5 mL of acetonitrile and dichloromethane (1:1, *v*/*v*) to extract non-sugar constituents from the nectar. The obtained organic extract was dried with anhydrous MgSO_4_, and the solvent evaporated under nitrogen gas before being reconstituted in 100 μL of methanol before HPTLC analysis.

#### 2.4.3. Honey

To prepare the unripe and mature clover honey extracts for HPTLC analysis, 1 g of each honey sample was dissolved in 2 mL of distilled water, followed by three subsequent extractions with 5 mL of acetonitrile and dichloromethane (1:1, *v*/*v*). The obtained organic extracts were dried with anhydrous MgSO_4_, and the solvent evaporated using nitrogen gas before being reconstituted in 100 μL of methanol before HPTLC fingerprinting.

### 2.5. HPTLC Analysis

The identification and quantification of isoflavone aglycones (i.e., biochanin A, formononetin, genistein and daidzein) in different clover samples was carried out using mobile phase A [26], while for glucosylated isoflavones (i.e., sissotrin, ononin, genistin and daidzin) mobile phase B was used [25]. 

Internal standard (THF for non-glycosidic compounds and naringin for glycosidic compounds) and all isoflavone standards were applied as a volume of 4 µL in the HPTLC analysis. Leaf (5 µL), flower bract (5 µL), flower nectar (15 µL), unripe honey (11 µL) and mature honey (9 µL) extracts were also applied. Standards and samples were applied as 8-mm bands positioned at 10 mm from the lower edge of the HPTLC plate at a rate of 150 nLs−1 using a semiautomated HPTLC application device (Linomat 5, CAMAG, Muttenz, Switzerland).

The chromatographic separation was performed on silica gel 60 F_254_ HPTLC plates (glass plates, 20 × 10 cm) in a saturated (33% relative humidity) automated development chamber (ADC2, CAMAG). The plates were pre-saturated with the mobile phase for 5 min, automatically developed to 80 mm for mobile phase A and 70 mm for mobile phase B at room temperature and dried for 5 min. The obtained chromatographic results were documented using a HPTLC imaging device (TLC Visualizer 2, CAMAG) at 254 nm. After derivatization with either VSA (3 mL, yellow nozzle, level 3) for the fingerprints obtained from mobile phase A or NP-PEG (3 mL NP, green nozzle, level 3 followed by 2 mL PEG, blue nozzle, level 2) for the fingerprints obtained from mobile phase B using a HPTLC derivatiser (CAMAG Derivatiser), the images were visualized at T white light and R366 nm. The chromatographic images were digitally processed and analyzed using specialized HPTLC software (visionCATS 3.1, CAMAG), which was also used to control the individual instrumentation modules.

The scanning of individual major bands in each sample was carried out using a TLC Scanner 4 in both UV-Vis mode (190–900 nm) and fluorescence mode (190–380 nm) before and after derivatization.

## 3. Results

### 3.1. Isoflavone Identification

**Figure 2:**
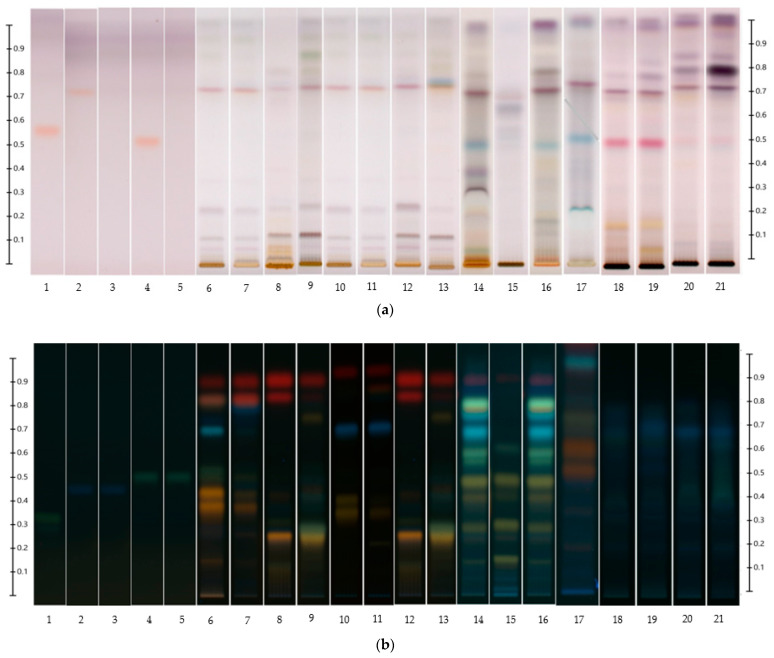
HPTLC fingerprints of (**a**) non-glycosidic isoflavones and clover extracts with mobile phase A at T white light after derivatizing with VSA; Track 1: THF; Track 2: Biochanin A; Track 3: Formononetin; Track 4: Genistein; Track 5: Daidzein; Track 6: NSE Leaves; Track 7: NFE Leaves; Track 8: Othello Leaves; Track 9: Electra Leaves; Track 10: NSE Flower Bracts; Track 11: NFE Flower Bracts; Track 12: Othello Flower Bracts; Track 13: Electra Flower Bracts; Track 14: NSE Flower Nectar; Track 15: NFE Flower Nectar Track 16: Othello Flower Nectar; Track 17: Electra Flower Nectar; Track 18: NSE Honey; Track 19: NFE Honey; Track 20: Othello Honey; and Track 21: Electra Honey and (**b**) glycosylated isoflavones and clover extracts with mobile phase B at 366 nm after derivatizing with NP-PEG. Track 1: Naringin; Track 2: Sissotrin; Track 3: Ononin; Track 4: Genistin; Track 5: Daidzin; Track 6: NSE Leaves; Track 7: NFE Leaves; Track 8: Othello Leaves; Track 9: Electra Leaves; Track 10: NSE Flower Bracts; Track 11: NFE Flower Bracts; Track 12: Othello Flower Bracts; Track 13: Electra Flower Bracts; Track 14: NSE Flower Nectar; Track 15: NFE Flower Nectar Track 16: Othello Flower Nectar; Track 17: Electra Flower Nectar; Track 18: NSE Honey; Track 19: NFE Honey; Track 20: Othello Honey; and Track 21: Electra Honey.

The identification of targeted compounds was performed using a validated HPTLC-derived phenolic compound database [27]. In brief, all the clover samples were first HPTLC fingerprinted under two different conditions, mobile phase A using derivatization with VSA (Figure 2a) and mobile phase B using derivatization with NP (Figure 2b). The resulting data (i.e., Rf values, colour hues, UV-Vis and fluorescence λmax prior to derivatization, UV-Vis and fluorescence λmax after derivatization) were compared with those of the standards [1,27]. Potential matches were confirmed by spectral overlay analysis using percent correlations on the basis of comparison of individual absorbance values [1,27]. The percent correlation of spectra matching is a crucial identification parameter: when this value approaches 100%, it indicates a stronger match between the spectra being compared. In this analysis, more than 90% correlation was found between bands of interest in the various clover samples and the corresponding isoflavone standards.

**Figure 3:**
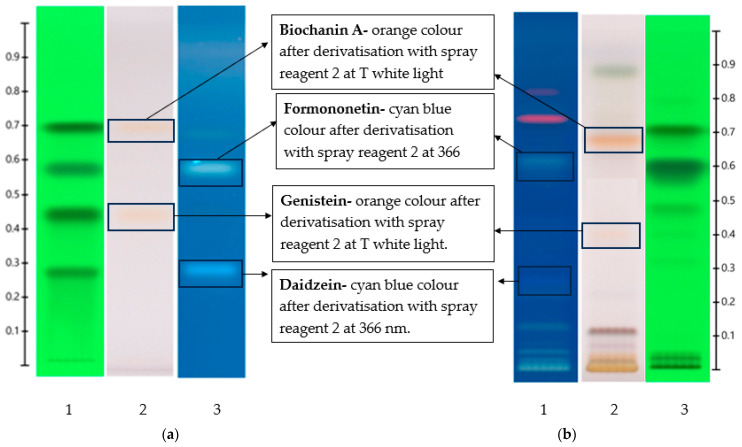
HPTLC-generated fingerprints using mobile phase A and derivatized using VSA; (**a**) fingerprints of isoflavone aglycones—Track 1: 254 nm after development, Track 2: T white after derivatization, Track 3: 366 nm after derivatization and (**b**) fingerprints of NFE leave extract—Track 1: 366 nm after derivatization, Track 2: T white after derivatization and Track 3: 254 nm after development.

**Figure 4:**
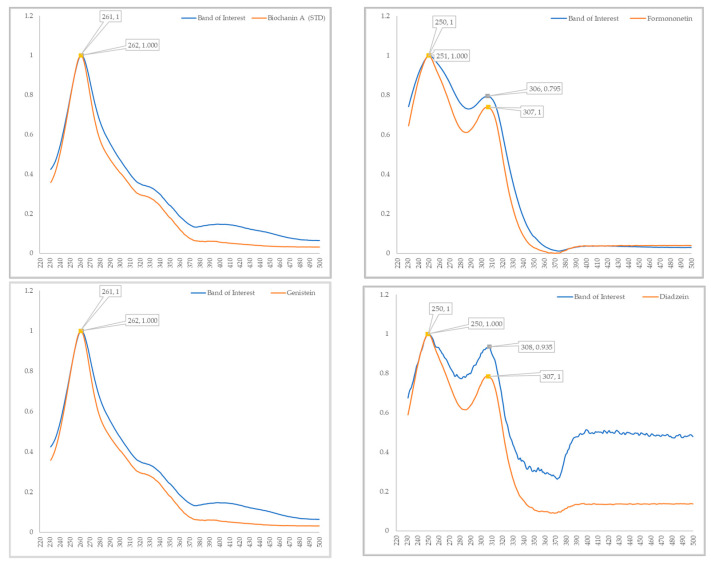
UV spectral overlay of the four unknown potential bands with their corresponding standards.

An example for the use of the database to match isoflavones in NFE red clover leave extract is shown in Figure 3. Four non-glycosidic isoflavones at Rf values 0.710, 0.589, 0.491 and 0.310, which corresponded to the Rf values of biochanin A (0.700), formononetin (0.600), genistein (0.460) and daidzein (0.290), respectively, were identified as potential matches using this methodology. The identification was confirmed by first comparing the hue values of the respective bands and their tentatively matched standards (Figure 3), followed by spectral matching. For this, the bands of interest in each sample were analysed using the TLC Scanner in both UV-Vis mode (190–900 nm) and fluorescence mode (190–380 nm). The resulting UV and fluorescence spectra were compared with those of the potential matches (see Figure 4 for UV spectral matches), and correlation percentages between the spectra calculated. The spectral matching shows % correlations of 99.7, 99.4, 99.5 and 94.8 between the band of interest and the corresponding reference standard biochanin A, formononetin, genistein and daidzein, respectively. This suggests a strong similarity between their spectral features, thus confirming the correct identification of these bands as biochanin A, formononetin, genistein and daidzein.

### 3.2. Isoflavone Quantification

Table 1 summarizes the identified non-glycosidic and glycosidic isoflavones in the various plant parts (i.e., leaves and flower bracts), the flower nectar and also the unripe and ripe honey of the four different clover species (two red clovers cultivars, NSE and NFE; the Sainfoin cultivar, Othello and the purple clover cultivar, Electra, respectively).

The same chromatographic instrumentation and parameters as described in Section 2.5 were employed in the quantification of the identified phytoestrogenic compounds in the various clover samples using previously published methodologies for aglyconic isoflavone [28] and for glucosylated isoflavone quantification. For this, standard curves of the respective isoflavone standards (20 µg/mL in methanol) were constructed by applying volumes of 3.0 to 15 µL (applied in 3 µL intervals), and each compound was quantified at its specific λmax using the evaluation feature of the VisionCATS 3.1 software [1,28]. Table 2 summarises the quantification of non-glycosidic and glycosidic isoflavones in the various plant parts (i.e., leaves and flower bracts), the flower nectar and the honey of the different clover species.

## 4. Discussion

In line with findings of others [12], this study confirms that the leaves of both red clover (*T. pratense*) cultivars act as a promising source of isoflavones. According to the literature [1,8,9,10,11,12], biochanin A and formononetin are the primary isoflavones found in red clover, particularly in its green aboveground parts (leaf and bracts of the flower head). Both compounds were also detected in this study in these plant extracts. Their levels were higher than those of other isoflavones across all investigated cultivars. As a novel finding of this study, these isoflavones were also detected and quantified in the flower nectar and also in the resulting ripe and unripe honeys. NSE clover was found to have the highest amount of biochanin A in its leaf and flower bract extracts, whereas formononetin was the major isoflavone found in NFE clover. This is not surprising, as these red clover cultivars were specifically bred for the pharmaceutical industry to express high levels of either of the two isoflavones. As expected, much lower concentrations of these isoflavones were detected in sainfoin and purple clover.

**Figure 5:**
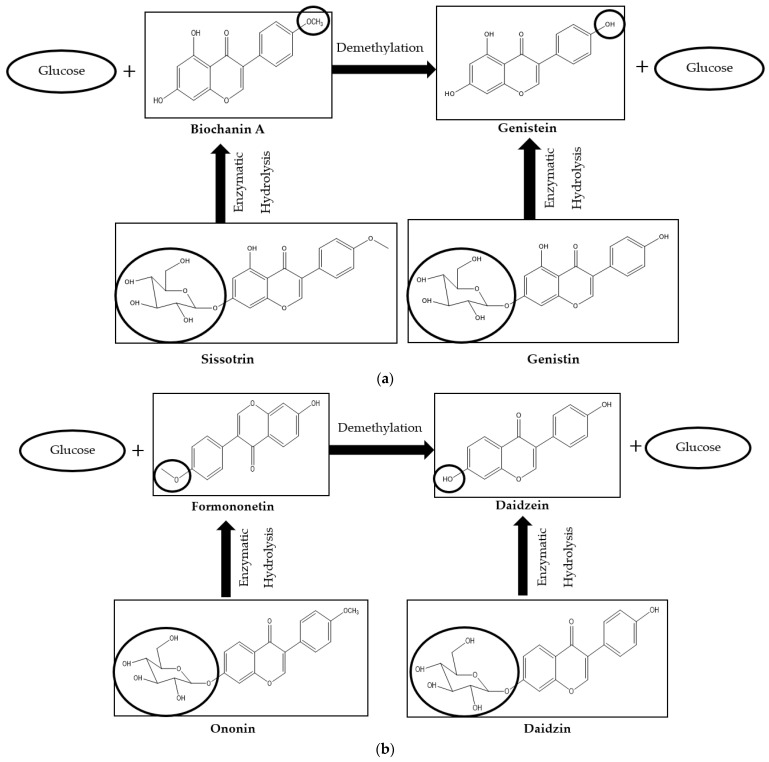
Biochemical conversion of isoflavones (**a**) biochemical pathway of genistein formation and (**b**) biochemical pathway of daidzein formation.

The pharmaceutical interest in red clover stems from its phytoestrogenic activity, which is related to the presence of isoflavones with structural similarity to the human female hormone 17-β-estradiol. They can therefore bind to both alpha and beta estrogen receptors, mimicking the action of estrogen in target organs. However, biochanin A and formononetin, found as predominant isoflavones in red clover leaves and flower head extracts, are methylated forms for genistein and daidzein, respectively, which express potent estrogenic activity. Both, the methylated isoflavones, biochanin A and formononetin as well as their demethylated form genistein and daidzein can also exist as glucosylated derivatives, known as sissotrin and ononin and as genistin and daidzin, respectively. On ingestion, the glucosidic forms are hydrolysed to their corresponding aglycones in the intestine and, following enzymatic demethylation, the active metabolites are formed (Figure 5).

**Figure 6:**
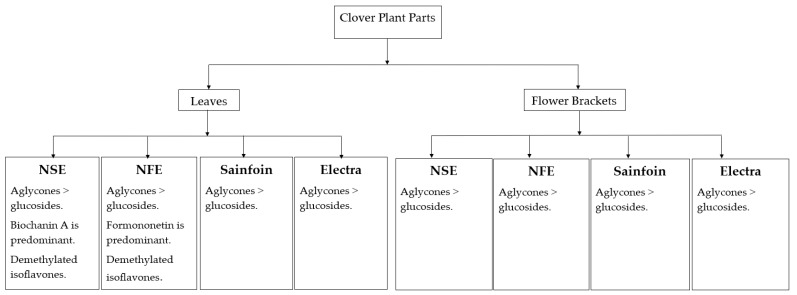
Isoflavones in clover plant components.

However, these biochemical conversions appear to also occur within the plants themselves as can be seen from the analysis of leave and flower bract extracts of the different clover species investigated in this study (Figure 6). Interestingly, only in the leaf extracts of both red clover cultivars were the demethylated isoflavones detected (alongside their corresponding methylated forms), but not in the other clover species, nor in any of the four flower bract extracts. Across the board, aglycones appear to be more prominent in these samples compared to their corresponding glucosylated derivatives.

Flower nectar serves as raw material for honey production. Honeybees gather nectar from blossoms and store it in their honey sac, which acts as their second stomach. There, enzymes break down the nectar’s sucrose content into fructose and glucose, which are more easily digestible monosaccharides. Enzymatic processes other than this biochemical conversion might also occur. Once the honey sac is full, bees return to their hive and pass the nectar mouth-to-mouth on to other bees. During this process, additional enzymes are added before the enzymatically processed nectar, which is known as unripe honey, is deposited into honeycombs. This unripe hive nectar is then turned into honey by the evaporation of its water content to around 20%, which is achieved by bees fanning their wings over the honeycombs. The process is complete when bees cap the honeycomb with wax to store the ripe (or mature) honey as a future food source. 

**Figure 7:**
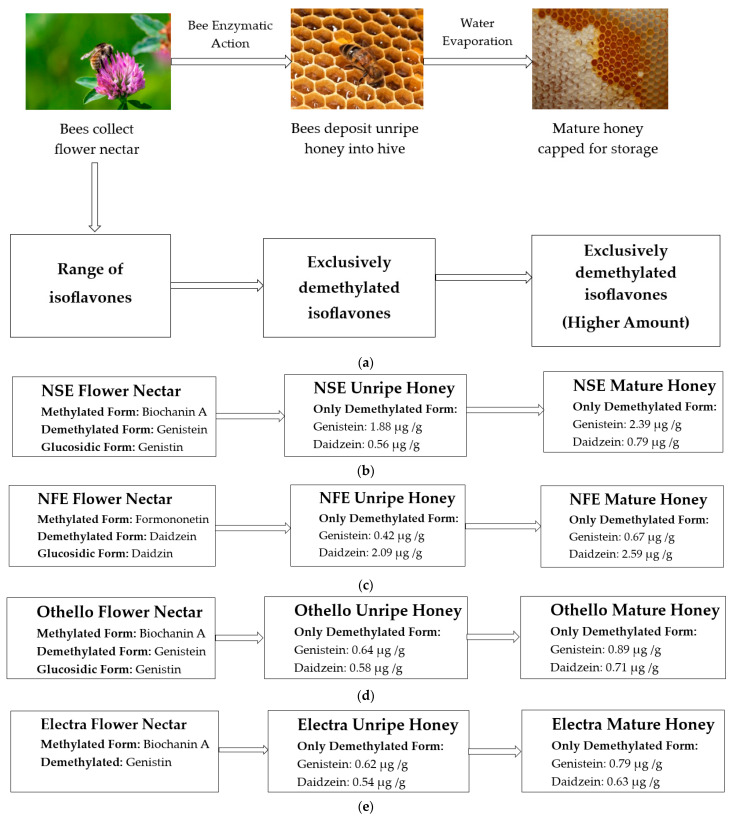
(**a**) Chemical conversion of isoflavones during transition from floral nectar to unripe honey and level of isoflavones in unripe and mature honey in the analysed clover cultivars; (**b**) NSE; (**c**) NFE; (**d**) Othello; and (**e**) Electra.

This study was able to demonstrate a fascinating interplay between flower nectar, honeybees and isoflavones, revealing intricate biochemical conversions in which honeybees appear to play a vital role (Figure 7a). Interestingly, most likely due to the activity of bee enzymes from the hypopharyngeal gland, all tested clover honeys (unripe and mature) were found to contain only demethylated form of isoflavones which are more potent among these eight analysed isoflavones, although in their corresponding flower nectars a range of isoflavones could be detected. It could also be demonstrated that no further chemical conversions take place once the unripe honey has been deposited into the honeycomb, as the unripe and ripe honeys were found to contain the same phytoestrogenic compounds, although at different concentrations. With the ripe honey being formed by removal of water from the corresponding unripe honey, genistein and daidzein are thus present at higher level expressed as per gram of sample of mature honey.

## 5. Conclusions

To assess the isoflavone profiles of flower nectar and honey from the four clover cultivars studied, plants were raised in enclosures, and their honeys directly harvested from nucleus beehives placed in these controlled environments. Through this approach, the production of monofloral honeys which reflect the unique characteristics of the respective clover flowers was aided. This study determined the unique isoflavonoid profile in various clover plant parts (i.e., leaves, flower bracts), flower nectar and unripe and ripe clover honeys. Both red clover cultivars were found to exclusively contain the more potent form of isoflavones, genistein and daidzein, along with their corresponding methylated precursors, biochanin A and formononetin in much higher concentrations than those detected in other clover species. 

This study shed light on intricate biochemical conversions by demonstrating a captivating interplay between flower nectar, honeybees and isoflavones, where honeybees seem to play a vital role. Despite differences in concentration, the phytoestrogenic isoflavone profiles detected in both unripe and ripe honeys were the same, which implies that no further chemical conversions take place when the unripe honey is deposited into the honeycomb and turns into mature honey. The isoflavone that was found to be most abundant in the respective red clover cultivar’s leaves and flower bracts was also identified as the dominant isoflavone in its flower nectar and honey. It is interesting to note that honeys harvested from Othello and Electra cultivars were also found to contain the demethylated form of isoflavones, although the corresponding flower nectars had different forms of isoflavones quantified. These findings demonstrate once more that the bioconversion of flower nectar constituents is facilitated by honeybee enzymes. 

This study demonstrates the presence of phytoestrogenic isoflavones in honey and connects them with their precursor isoflavones in the respective flower nectars. While the wide range of health benefits associated with various clovers is still being explored worldwide, the estrogenic properties established for red clover plant parts, and with this study also its honey, make red clover an intriguing natural remedy. However, the levels of isoflavones in red clover derived honeys are lower compared to those detected in its plant parts (e.g., leaves and flower bracts). The recommended daily intake of isoflavones for the management of menopausal symptoms is 40–80 mg/day which might not be achievable solely by taking red clover honey. However, there could still be an opportunity to develop red clover honey or other nectar products into health foods or dietary supplements given the documented presence of phytoestrogenic isoflavones.

## Figures and Tables

**Table 1:** Identified isoflavones in clover leaves, flower bracts, flower nectar and honey.

Cover Sample		Non-Glycosidic Isoflavones	Glycosidic Isoflavones
NSE	Leaves	Daidzein, genistein, formononetin, biochanin A	Genistin, daidzin
	Flower bracts	Daidzein, genistein, formononetin, biochanin A	Genistin, daidzin
	Flower nectar	Biochanin A, genistein	Genistin
	Unripe and ripe honey	genistein, daidzein	None found
NFE	Leaves	Daidzein, genistein, formononetin, biochanin A	Genistin, daidzin
	Flower bracts	Daidzein, genistein, formononetin, biochanin A	Genistin, daidzin
	Flower nectar	Formononetin, daidzein	Daidzin
	Unripe and ripe honey	genistein, daidzein	None found
Othello	Leaves	Biochanin A, formononetin	Sissotrin, ononin
	Flower bracts	Biochanin A, formononetin	Genistin, ononin
	Flower nectar	Biochanin A, genistein	Genistin
	Unripe and ripe honey	genistein, daidzein	None found
Electra	Leaves	Biochanin A, formononetin	Sissotrin, ononin
	Flower bracts	Biochanin A, formononetin	Genistin, ononin
	Flower nectar	Biochanin A	Genistin
	Unripe and ripe honey	Genistein, daidzein	None found

**Table 2:** Isoflavones levels in components of the four clovers.

Clover Species	Part	Non-Glycosidic Isoflavones (µg/gm)			Glycosidic Isoflavones (µg/gm)			Total (Non-Glycosidic and Glycosidic) Isoflavones (µg/gm)
		Isoflavone	Individual Amount	Total	Isoflavone	Individual Amount	Total	
NSE	Leaves	Biochanin A	5800.00	9049.00	Genistin	16.00	24.00	9073.00
		Formononetin	2100.00					
		Genistein	1000.00		Daidzin	6.00		
		Daidzein	149.00					
	Flower bracts	Biochanin A	1210.00	2490.00	Genistin	2.00	2.49	2492.49
		Formononetin	1280.00		Daidzin	0.49		
	Flower nectar	Biochanin A	3.00	3.22	Genistin	0.26	0.26	3.48
		Genistein	0.22					
	Unripe honey	Genistein	1.88	2.44	Not found			2.44
		Daidzein	0.56					
	Honey	Genistein	2.39	3.18	Not found			3.18
		Daidzein	0.79					
NFE	Leaves	Biochanin A	2800.00	12,043.00	Genistin	2.00	23.00	12,066.00
		Formononetin	9100.00					
		Genistein	91.00		Daidzin	21.00		
		Daidzein	52.00					
	Flower bracts	Biochanin A	1000.00	13,000.00	Genistin	0.39	3.41	13,003.41
		Formononetin	12,000.00		Daidzin	3.20		
	Flower nectar	Formononetin	4.80	5.23	Daidzin	0.43	0.43	5.66
		Daidzein	0.43					
	Unripe honey	Genistein	0.42	2.51	Not found			2.51
		Daidzein	2.09					
	Honey	Genistein	0.67	3.26	Not found			3.26
		Daidzein	2.59					
Othello	Leaves	Biochanin A	33.00	84.00	Sissotrin	0.78	1.92	85.92
		Formononetin	51.00		Ononin	1.14		
	Flower bracts	Biochanin A	27.00	75.00	Genistin	1.67	1.79	76.67
		Formononetin	48.00		Ononin	0.12		
	Flower nectar	Biochanin A	1.49	1.7	Genistin	0.17	0.17	1.87
		Genistein	0.21					
	Unripe honey	Genistein	0.64	1.22	Not found			1.22
		Daidzein	0.58					
	Honey	Genistein	0.89	1.6	Not found			1.6
		Daidzein	0.71					
Electra	Leaves	Biochanin A	236.00	379.00	Sissotrin	1.00	2.20	381.20
		Formononetin	143.00		Ononin	1.20		
	Flower bracts	Biochanin A	39.00	90.00	Genistin	1.10	1.63	91.63
		Formononetin	51.00		Ononin	0.53		
	Flower nectar	Biochanin A	1.22	1.22	Genistin	0.21	0.21	1.43
	Unripe honey	Genistein	0.62	1.16	Not found			1.16
		Daidzein	0.54					
	Honey	Genistein	0.79	1.42	Not found			1.42
		Daidzein	0.63					

## Data Availability

The original contributions presented in the study are included in the article, further inquiries can be directed to the corresponding author.

## References

[1] Sultana S., Foster K., Bates T., Hossain M.L., Lim L.Y., Hammer K., Locher C. (2024). Determination of Physicochemical Characteristics, Phytochemical Profile and Antioxidant Activity of Various Clover Honeys. Chem. Biodivers..

[2] Pellett F.C. (1947). American Honey Plants.

[3] Ogle D., Cane J., Fink F., St. John L., Stannard M., Dring T. (2007). Plants for Pollinators in the Intermountain West.

[4] Abell D.C., Friebe H., Schweger C., Kwok A.S.K., Sporns P. (1996). Comparison of processed unifloral clover and canola honey. Apidologie.

[5] Oertel E. (1967). Beekeeping in the United States; United States Department of Agriculture, p. 16. Archived from the Original on 2023-01-16. https://scientificbeekeeping.com/scibeeimages/USDA-1967-Beekeeping-in-the-United-States.

[6] Sultana S., Foster K., Lim L.Y., Hammer K., Locher C. (2022). A Review of the Phytochemistry and Bioactivity of Clover Honeys (*Trifolium* spp.). Foods.

[7] Mostrom M., Evans T.J., Gupta R.C. (2012). Phytoestrogens. Veterinary Toxicology—Basic and Clinical Principles.

[8] Francis C.M., Millington A., Bailey E. (1967). The Distribution of Oestrogenic Isoflavones in the Genus Trifolium. Aust. J. Agric. Res..

[9] Klejdus B., Vitamvásová-Štěrbová D., Kubáň V. (2001). Identification of Isoflavone Conjugates in Red Clover (*Trifolium pratense*) by Liquid Chromatography–Mass Spectrometry after Two-Dimensional Solid-Phase Extraction. Anal. Chim. Acta.

[10] de Rijke E., Zappey H., Ariese F., Gooijer C., Brinkman U.A.T. (2004). Flavonoids in Leguminosae: Analysis of Extracts of *T. pratense* L., *T. dubium* L., *T. repens* L., and *L. corniculatus* L. Leaves Using Liquid Chromatography with UV, Mass Spectrometric and Fluorescence Detection. Anal. Bioanal. Chem.

[11] Lemežienė N., Padarauskas A., Butkutė B., Cesevičienė J., Taujenis L., Norkevičienė E. (2015). The Concentration of Isoflavones in Red Clover (*Trifolium pratense* L.) at Flowering Stage. Zemdirb. Agric..

[12] Mikulić M., Atanacković Krstonošić M., Kladar N., Vasiljević S., Katanski S., Mamlić Z., Rakić D., Cvejić J. (2024). Phytochemical Composition of Different Red Clover Genotypes Based on Plant Part and Genetic Traits. Foods.

[13] Mostrom M., Evans T.J. (2011). Phytoestrogens Reproductive and Developmental Toxicology.

[14] Butkutė B., Lemežiene N., Dabkevičienė G., Jakštas V., Vilčinskas E., Janulis V. (2014). Source of Variation of Isoflavone Concentrations in Perennial Clover Species. Pharmacogn. Mag..

[15] Brandli A., Simpson J.S., Ventura S. (2010). Isoflavones Isolated from Red Clover (*Trifolium pratense*) Inhibit Smooth Muscle Contraction of the Isolated Rat Prostate Gland. Phytomedicine.

[16] Wu Q., Wang M., Simon J.E. (2003). Determination of Isoflavones in Red Clover and Related Species by High-Performance Liquid Chromatography Combined with Ultraviolet and Mass Spectrometric Detection. J. Chromatogr. A.

[17] Sterbova D., Stratil P., Kuban V. (2003). Identification and characterization of isoflavones in plant material by HPLC-DAD-MS tandem. WKlejdus. B Chem. Listy.

[18] Kaurinovic B., Popovic M., Vlaisavljevic S., Schwartsova H., Vojinovic-Miloradov M. (2012). Antioxidant Profile of *Trifolium pratense* L.. Molecules.

[19] Mace T.A., Ware M.B., King S.A., Loftus S., Farren M.R., McMichael E., Scoville S., Geraghty C., Young G., Carson W.E. (2019). Soy Isoflavones and Their Metabolites Modulate Cytokine-Induced Natural Killer Cell Function. Sci. Rep..

[20] Hsiao Y.H., Ho C.T., Pan M.H. (2020). Bioavailability and Health Benefits of Major Isoflavone Aglycones and Their Metabolites. J. Funct. Foods.

[21] Křížová L., Dadáková K., Farková V., Jafari S.M., Rashidinejad A., Simal-Gandara J. (2022). Isoflavones. Handbook of Food Bioactive Ingredients.

[22] Křížová L., Dadáková K., Kašparovská J., Kašparovský T. (2019). Isoflavones. Molecules.

[23] Corbet S.A. (2003). Nectar Sugar Content: Estimating Standing Crop and Secretion Rate in the Field. Apidologie.

[24] Morrant D.S., Schumann R., Petit S. (2009). Field Methods for Sampling and Storing Nectar from Flowers with Low Nectar Volumes. Ann. Bot..

[25] Sultana S., Foster K., Hossain M.L., Lim L.Y., Locher C. (2023). Development and Validation of an Assay for the Quantification of Glycosides Using High-Performance Thin-Layer Chromatography (HPTLC). JPC-J Planar Chromat..

[26] Wójciak-Kosior M., Sowa I., Strzemski M., Rokicka K., Tomasz Blicharski T., Bogucka-Kocka A. (2014). Application of High-Performance Thin Layer Chromatography for Qualitative Analysis of Isoflavones in Medicinal Plants. Int. J. Res. Pharm. Chem..

[27] Lawag I.L., Sostaric T., Lim L.Y., Hammer K., Locher C. (2022). The Development and Application of A HPTLC-Derived Database for the Identification of Phenolics in Honey. Molecules.

[28] Lawag I.L., Islam M.K., Sostaric T., Lim L.Y., Hammer K., Locher C. (2023). Antioxidant Activity and Phenolic Compound Identification and Quantification in Western Australian Honeys. Antioxidants.

